# Role of liver sinusoidal endothelial cell in metabolic dysfunction-associated fatty liver disease

**DOI:** 10.1186/s12964-024-01720-9

**Published:** 2024-06-28

**Authors:** Qiongyao He, Wu He, Hui Dong, Yujin Guo, Gang Yuan, Xiaoli Shi, Dingkun Wang, Fuer Lu

**Affiliations:** 1 Institute of Integrated Traditional Chinese and Western Medicine, Tongji Hospital, Tongji Medical College, Huazhong University of Science and Technology, Wuhan, 430030 China; 2 Division of Cardiology, Tongji Hospital, Tongji Medical College, Huazhong University of Science and Technology, Wuhan, 430030 China; 3 Department of Integrated Traditional Chinese and Western Medicine, Tongji Medical College, Tongji Hospital, Huazhong University of Science and Technology, Wuhan, 430030 China; 4 Department of Endocrinology, Department of Internal Medicine, Tongji Hospital, Tongji Medical College, Huazhong University of Science and Technology, Wuhan, 430030 China; 5 Hubei Key Laboratory of Genetics and Molecular Mechanisms of Cardiological Disorders, Wuhan, 430030 China

**Keywords:** Capillarization, Liver sinusoidal endothelial cells, Endothelial dysfunction, Angiogenesis, Steatosis

## Abstract

Liver sinusoidal endothelial cells (LSECs) are highly specialized endothelial cells that represent the interface between blood cells on one side and hepatocytes on the other side. LSECs not only form a barrier within the hepatic sinus, but also play important physiological functions such as regulating hepatic vascular pressure, anti-inflammatory and anti-fibrotic. Pathologically, pathogenic factors can induce LSECs capillarization, that is, loss of fenestra and dysfunction, which are conducive to early steatosis, lay the foundation for the progression of metabolic dysfunction-associated fatty liver disease (MAFLD), and accelerate metabolic dysfunction-associated steatohepatitis (MASH) and liver fibrosis. The unique localization, phenotype, and function of LSECs make them potential candidates for reducing liver injury, inflammation, and preventing or reversing fibrosis in the future.

## Background

Despite the substantial gains in our understanding of non-alcoholic fatty liver disease (NAFLD)/non-alcoholic steatohepatitis (NASH) over the past 2 decades, there has been some dissatisfaction with the terminology “non-alcoholic” which overemphasizes “alcohol” and underemphasizes the root cause of this liver disease, namely, the predisposing metabolic risk factors. As a potential remedy, a name change from NAFLD to MAFLD has been proposed [1] and endorsed by international expert consensus [2–4]. MAFLD affects about a quarter of the world’s adult population [2, 5, 6]. The subtype of MAFLD can be characterized as MASH, which is a potentially progressive liver disease that can lead to liver cirrhosis and hepatocellular carcinoma (HCC) [7–9]. MAFLD is associated with obesity, insulin resistance and other metabolic abnormalities, collectively referred to as metabolic syndrome [10–12]. Current views on the pathogenesis of MAFLD focus on the response of hepatocytes to insulin resistance and lipotoxicity, with immune system and HSCs activation considered secondary events [7].

Hepatic microcirculation environment is mainly composed of LSECs, hepatic stellate cells (HSCs) and Kupffer cells (KCs) [13, 14]. Hepatocytes are arranged in hexagonal lobules, separated from the thin-walled LSECs by the Disse space, where HSCs is located [15]. LSECs assemble hepatic sinusoids, which are characterized by the lack of basement membrane (BM), open fenestration and no diaphragms, forming a permeable barrier [16, 17]. Monocyte-derived resident macrophages, known as KCs, reside in the hepatic sinusoid and are the first line of defense of the liver immune system [18]. LSECs represent the interface between blood and other liver tissues, accounting for about 15 to 20% of the number of hepatocytes, but its role in liver disease has not yet been elucidated and valued [19]. This Review focuses on the physiological function of LSECs and its role in the pathological progress of MAFLD.

## The research process of LSECs

**Fig. 1:**
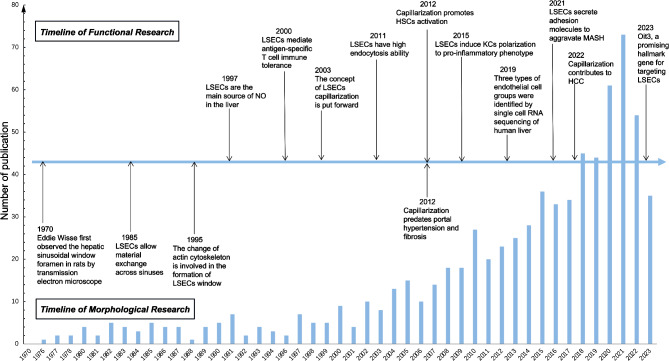
Schedule of LSECs morphology and function research. The graph shows the main findings of LSECs in the progress of MAFLD. The line chart reflects the total number of publications targeting LSECs in recent years. The research content pointed by the arrows provide a basis for us to understand the morphological structure (lower box) and physiological or pathological function of LSECs (upper box). In the past few decades, publications in this field have rapidly increased, and some targets related to LSECs have entered clinical research through basic trials, all of which are discussed in more detail in this text

In 1970, Eddie Wisse first observed the rat hepatic sinusoidal fenestration with a transmission electron microscope [20]. This new visualization enabled LSECs to differentiate from other types of cells, including KCs and other vascular endothelial cells [21]. At that time, it was thought that the major part of the transport and exchange of fluid, solutes and particles between the blood and the space of Disse occurred through these open fenestrae [22], that was, the liver was the central organ for lipoprotein metabolism [23]. And the mainstream view was that the change of actin cytoskeleton might play a key role in development of fenestrae [24]. In 1997, Shah et al. proved that LSECs were the main source of liver NO, so they were involved in the regulation of hepatic vascular pressure [25]. In 2000 and beyond, Limmer et al. found that LSECs had immunomodulatory function, that was, they mediated lymphocyte recruitment to the liver and inhibited inflammatory T cell activity to form immune tolerance to circulating soluble antigens [26, 27].These findings laid a solid foundation for subsequent research. In 2003, the concept of capillarization was proposed [28], and this pathological change of LSECs could induce KCs polarization to pro-inflammatory phenotype [29] and HSCs activation, accelerating the progression of MAFLD [15]. MAFLD was closely related to the balance of lipid metabolism, it was then observed that in addition to the LSECs fenestration, the high endocytosis of LSECs was also beneficial to the rapid removal of blood borne ligands, thus maintaining lipid homeostasis [30], and the loss of LSECs transport function could accelerate disease progression. In recent years, LSECs have been gradually discovered to be involved in the progression of many other liver diseases, such as MASH [31], liver fibrosis [15] and HCC [32] by regulating fenestration, secretion of adhesion molecules and vascular secretion signals [33], in which fenestration loss was recognized to be the earliest event [15]. Nowadays, the number of studies on LSECs has increased dramatically, and the research has become more rigorous and meticulous. In 2019, three different groups of endothelial cells were identified by single cell RNA sequencing of human liver, which laid a foundation for future subgroup research and functional refinement of hepatic endothelium [34]. In addition, LSECs specific marker genes have not been introduced before, and the latest research has found that Oit3 was a promising marker gene targeting LSECs, providing a valuable model for studying the complexity of LSECs in liver diseases [35] (Fig. 1).

## Unique features of LSECs

### LSECs form blood sinuses with fenestrations

The diameters of sinusoidal fenestrations in normal individuals are between 107+/-1.5 nm [36], and the diameters along the sinusoidal direction vary with oxygen concentration [37]. Fenestration is not unique to hepatic endothelial cells, but also seen in endothelial cells of other organs, such as pancreas [38], kidney [39], spleen [40], bone marrow [41], and even tumor vascular system [42]. However, unlike other endothelial cell groups, liver fenestration lacks a septum or basal layer and is grouped into organized sieve plates, making LSECs highly permeable [39]. Dietary lipids present in circulation must be transported through the hepatic sinusoids for tissue metabolism [43].LSECs are the main channel for two-way lipid exchange between blood and liver parenchyma, allowing the effective transfer of lipoproteins, chylous particle residues and other macromolecules from sinusoidal blood to the Disse space, where they are absorbed by hepatocytes [23, 44]. Secondly, LSECs regulate lipid transfer through its high endocytosis, from cellular components such as collagen and hyaluronic acid to acetylated low-density lipoprotein (LDL) cholesterol, immune complexes and exogenous antigens to maintain the balance of lipid metabolism in the liver [45, 46].

### LSECs capillarization

Capillarization is characterized by deposition of ectopic BM, formation of continuous microvascular endothelial cell layer and increased expression of VE-cadherin, which are the initial pathological changes related to MAFLD [47]. The triggers of capillarization have not been fully determined, but excessive dietary micronutrients (including lipids, carbohydrates and intestinal microbial derivatives) may play a role. A study on the addition of 25 different macronutrients and energy to 15-month-old mice showed that the distribution of macronutrients was related to the change of fenestration, porosity was negatively correlated with dietary fat intake, and the fenestration diameter was negatively correlated with protein or carbohydrate intake [48]. In vitro studies have shown that fenestration exclusion occurs after excessive lipid exposure. For example, exposure of human primary LSECs to oxidized low-density lipoprotein (ox-LDL) increased the lectin-like ox-LDL receptor 1 expression at both the mRNA and protein levels in a dose- and time-dependent manner. Ox-LDL stimulation increased reactive oxygen species (ROS) generation and NF-κB activation, upregulated endothelin-1 (ET-1) and caveolin 1 expression, downregulated endothelial nitric oxide synthase (eNOS) expression and reduced the fenestra diameter and porosity [49]. Fenestration is also related to the metabolic changes of intestinal microorganisms caused by diet. The changes of several fatty acid levels (C16:0, C19:0 and C20:4) caused by the higher abundance of Firmicutes and reduced abundance of Bacteroidetes are significantly negatively correlated with the number of fenestrations [48]. In addition, lipopolysaccharide (LPS) may also play a role in inducing the arrangement of fenestrations. A study of rats injected intravenously with LPS for 7 days showed that the diameter and number of fenestrations decreased, and the porosity could be reduced to 40% of the control group [50].

**Fig. 2:**
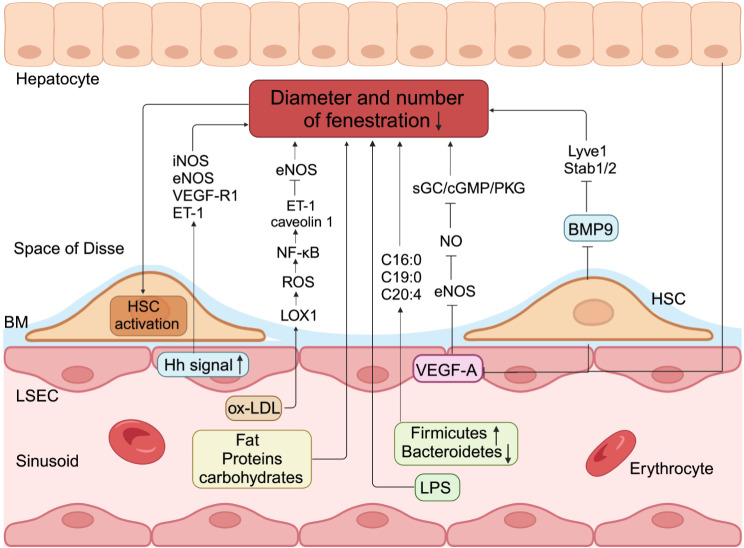
The pathological mechanism of capillarization in LSECs. Excessive intake of dietary fat, protein or carbohydrates, as well as changes of intestinal microorganisms or LPS can induce a decrease in the diameter and number of LSECs fenestrations. In addition, ox-LDL exposure increased the lectin-like ox-LDL receptor 1 expression at both the mRNA and protein levels, increased ROS generation and NF-κB activation, and then upregulated ET-1 and caveolin 1 expression, downregulated eNOS expression, thus reducing the fenestra diameter and porosity. Abnormal activation of Hh signal in LSECs is accompanied by increased expression of iNOS, eNOS, VEGF-R1 and ET-1, as well as LSECs capillarization in vitro. Equally important, when the disruption of the local endocrine environment leads to a decrease in the secretion of VEGF-A by hepatocytes and HSCs, NO downstream of eNOS induces a significant closure of the LSECs fenestration through the sGC/cGMP/PKG pathway. Lastly, decreased secretion of BMP9 generated by HSCs also significantly increases the number of basal layer deposition and reduces number of fenestrae by downregulating LSECs terminal differentiation markers. This capillarization of LSECs is accompanied by the loss of gatekeeper function, which leads to the activation of HSCs

In addition to the phenotypic changes induced by diet, the fenestration arrangement phenomenon may also be related to the signal changes of LSECs itself or crosstalk between the surrounding cells. Hedgehog (Hh) signaling is an important component in the regulation of vasculogenesis, which increases during liver injury and influences the function of liver cells including cholangiocytes, hepatocytes, HSCs and LSECs [51, 52]. The abnormal activation of Hh signal in LSECs is accompanied by the capillarization that was associated with increased expression of inducible nitric oxide synthase (iNOS), eNOS, vascular endothelial growth factor (VEGF)-R1 and ET-1 in vitro [53]. Inhibition of Hh pathway in vivo and in vitro can alleviate early steatosis and fibrosis of liver by promoting fenestration recovery, helping to clear plasma chylous particle residues and antagonizing HSCs activation [54]. In addition, there is evidence that LSECs and HSCs maintain each other’s phenotypic differentiation. LSECs fenestration is maintained by NO downstream of VEGF-A secreted by hepatocytes or HSCs [16, 55, 56]. VEGF-A stimulates NO release from eNOS in LSECs. NO in turn acts through soluble guanylate cyclase (sGC), conversion of guanosine triphosphate to cyclic guanosinc monophosphate (cGMP), and stimulation of protein kinase G (PKG), which can then phosphorylate protein targets. In addition to the VEGF-A-stimulated NO pathway, maintenance of the LSECs phenotype also requires an NO-independent pathway, which remains to be characterized [15]. When VEGF-A/NO secretion decreases, the LSECs fenestration closes significantly and the gatekeeper function is lost, indicating that VEGF-A signaling plays a crucial role in maintaining LSECs differentiation [15]. Bone morphogenetic protein 9 (BMP9) is another circulating factor produced by HSCs, which plays a key role in vascular quiescence. In BMP9 knockout mice, the expression of LSECs terminal differentiation markers (Lyve1, Stab1, Stab2, Ehd3, Cd209b, eNOS, MAF, PLVAP) decreased, the number of basal layer deposition increased and permeable fenestrations decreased significantly [57] (Fig. 2).

### Hepatic blood flow regulation

The hepatic sinusoid has a dual blood supply, receiving blood flow from the portal vein (70%) and the hepatic artery (30%) [17]. Blood pressure is balanced in the sinuses and the blood is drained to the hepatic vein and inferior vena cava [58]. The circadian rhythm of hepatic blood flow caused by digestion changes significantly, but the pressure gradient of hepatic vein in normal individuals is kept at 4mmHg or lower, demonstrating the precise regulation of hepatic vascular tension [59]. Intrahepatic shear stress is considered to be the main driving factor of hepatic blood flow regulation [60]. In the liver, as in other vascular beds, endothelial cells can produce vasodilators in response to increased shear stress to lower blood pressure [61]. LSECs are the main source of NO in normal liver, which is generated through shear stress activated eNOS [25]and induce downregulation of vasoconstrictor molecules, including ET-1 [62]. Other molecules released by LSECs that regulate blood flow include the vasodilator CO and metabolites of cyclooxygenase (COX) pathway (thromboxane A2, prostacyclin) [63], all of which act paracrine on HSCs in the Disse space, causing blood pressure to drop. Unlike most vascular beds, blood flow is mainly regulated by smooth muscle cells, which play a limited role in the liver because they are rarely present in the portal vein [64]. Hepatic blood flow regulation is mainly achieved through the contraction of the tentacle-like structure of perivascular HSCs. Healthy LSECs keep HSCs at rest, thus inhibiting its vasoconstriction [65].

### Cell interaction and immune regulation

The sieve pore properties of sinusoidal endothelium expose the liver to microbes and food antigens that constantly come from the gastrointestinal tract through the portal vein [66–68]. The liver needs to ensure that it does not cause excessive inflammatory activation while eliminating invasive pathogens [69–71]. The first site of exposure to these antigens occurs in the hepatic sinusoid. KCs and LSECs are important participants in absorbing and eliminating soluble antigens entering through the portal vein and in determining the nature of any immune response triggered by such antigens [39, 72]. LSECs induce CD4 T cell tolerance through direct cell contact, which leads to interferon-γ or interleukin-17 release inhibition. This lasting inhibition depends on the interaction of co-stimulatory molecules (CD40, CD80, CD86), major histocompatibility complex (MHC) II molecules and programmed death ligand 1 (PD-L1) on LSECs with programmed death 1 (PD-1) and other receptors on CD4 T cells, and is induced by IL-10 abundantly produced in the liver, e.g., by KCs or HSCs [27]. In addition, the interaction between LSECs and CD8 T cells can also induce liver immune tolerance. Using bone-marrow chimeras and a novel transgenic mouse model (Tie2-H-2 K(b) mice) with endothelial cell-specific MHC I expression, it was found that LSECs preferentially absorbed systemically circulating antigen, resulting in cross-presentation on MHC class I molecules, which in turn led to rapid retention and cellular tolerance of antigen-specific native CD8 T cells in the liver [73]. Using electron microscopy, a study demonstrated that liver resident lymphocytes as well as circulating native CD8 T cells made direct contact with hepatocytes through cytoplasmic extensions penetrating the endothelial fenestrations that perforate the LSECs [74]. This unique interaction is significant in inducing liver immune tolerance. However, there is a dynamic regulation of LSECs on the induction of tolerance to CD8 T cells. Although LSECs cross-presentation at low-antigen concentrations resulted in tolerance, they induced CD 8 T cells differentiated into effector T cells at high-antigen concentrations or upon viral infection of LSECs, which can induce the activation of liver immune response [75, 76]. Meanwhile, LSECs also play a certain role in eliminating translocating gut bacteria [71]. KCs are important protective barriers as they engulf translocated microbial products and live bacteria with the promotion of commensal-derived D-lactic acid [77]. LSECs perceive microorganisms and allow KCs and natural killer T cells to locate the area around the portal vein as entry points for invading microorganisms [78]. This immune zonation in the liver protects against translocating gut bacteria [78]. These results highlight specific mechanisms by which LSECs governs the balance between tolerance and immunity.

### Maintain HSCs quiescence

The dedifferentiation of LSECs plays a key role in the process of HSCs activation and fibrosis [79–81]. Activation of HSCs, which transdifferentiates from resting, vitamin A storage cells into proliferative, fibrotic myofibroblasts, has now been identified as a major driver of fibrosis in mice and human liver injury [82]. In vitro studies have shown that differentiated LSECs can prevent HSCs activation and promote the return of activated HSC (aHSC) to quiet HSC (qHSC) through VEGF-A-stimulated NO production, but LSECs will lose this effect during dedifferentiation or capillarization [65, 83],

## The role of LSECs during hepatic steatosis

The capillarization of LSECs occurs in the very early stage of MAFLD, before the establishment of steatosis [84, 85], and before the appearance of activated KCs and HSCs [29]. The phenotypic changes of LSECs promote the development of liver steatosis by preventing the release of very-low-density lipoprotein (VLDL) from hepatocytes to the hepatic sinusoid, resulting in an increase in the storage of total cholesterol (TC) and triglycerides (TG) in the liver [84].In addition, by preventing chylous particle residues into hepatocytes, it leads to a significant increase in plasma cholesterol triglyceride and LDL levels, as well as the production of de novo lipids in the liver, which promote hyperlipidemia and early pathological changes of MAFLD [86].

Previous studies have shown that severe steatosis without inflammation or fibrosis can induce portal hypertension and hyperdynamic circulation, accompanied by intrahepatic vascular hyperreactivity and extrahepatic vascular hyporeactivity [87]. At the same time, the increase of intrahepatic vascular resistance driven by vascular dysfunction may also promote the progress of MAFLD through intralobular hypoxia, and the increase of portal pressure is positively correlated with the severity of steatosis [87–89]. It has been determined that the mechanisms leading to increased portal resistance include mechanical factors, which are a direct result of fibrosis deposition, and other factors associated with endothelial dysfunction, insufficient production of NO in the liver, increased production of vasoconstrictors, changes in microvascular structure and increased contraction of HSCs [90–94].

**Fig. 3:**
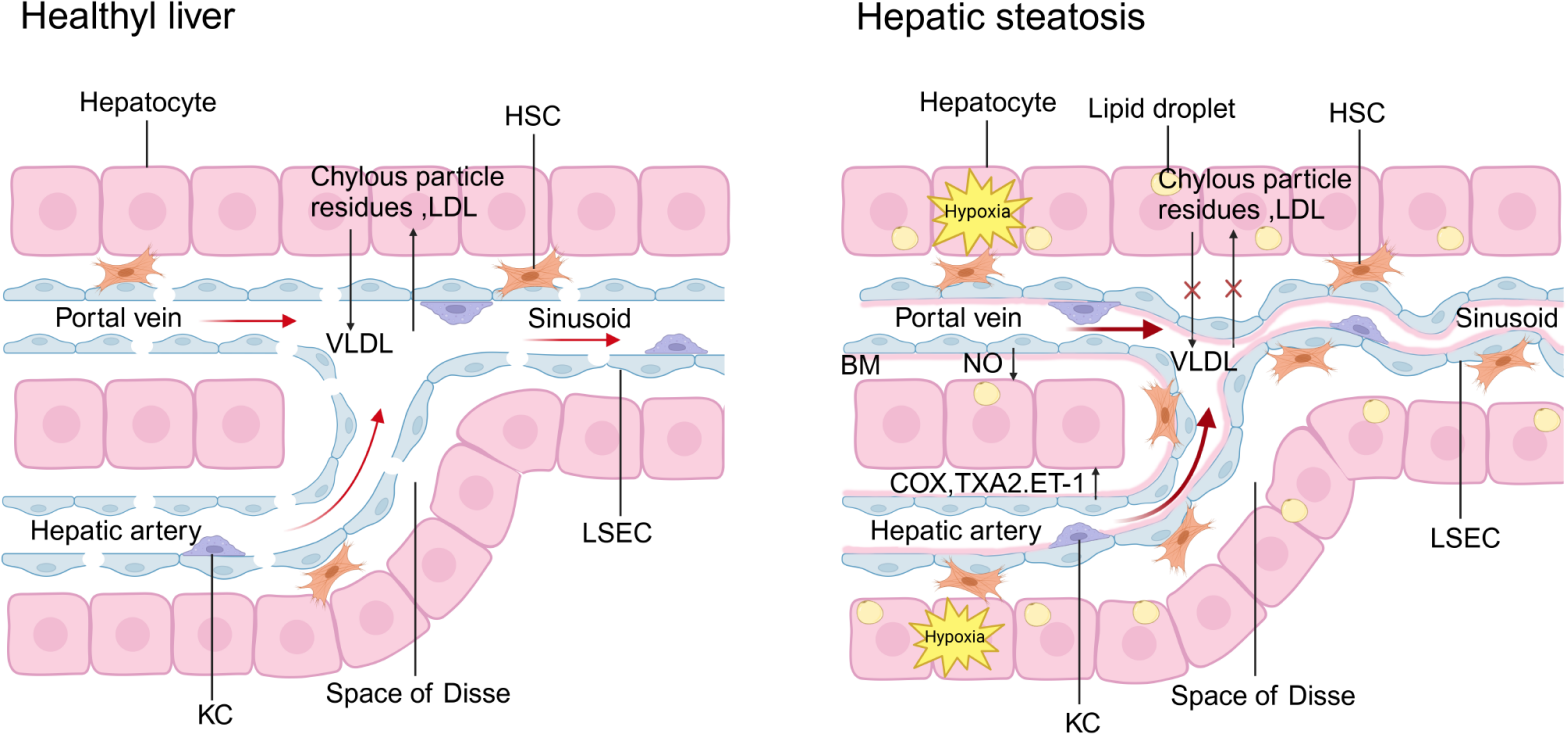
LSECs related pathological mechanism of hepatic steatosis. Normal hepatic sinusoids receive dual blood flow from the portal vein and hepatic artery, and the fenestration of hepatic sinusoids allows the absorption of TC, TG and LDL from the bloodstream into hepatocytes, as well as the release of VLDL from hepatocytes to the bloodstream. However, in the very early stage of MAFLD, the phenotypic changes of LSECs cause an increase in the storage of TC and TG in the liver by preventing the release of VLDL from hepatocytes and by preventing chylous particle residues and LDL into hepatocytes, leading to hyperlipidemia and hepatic steatosis. At the same time, insufficient NO production and increased production of COX, TXA2 and ET-1 in the liver lead to hemodynamic changes and increased vasoconstriction, which induce intralobular hypoxia to promote the progression of MAFLD. Equally important, the hepatic microvascular structure of steatosis changes significantly and the parallel sinusoidal waves are replaced by completely irregular and flat vascular patterns. HSCs recruited into the Disse space aggravates endothelial dysfunction and portal hypertension by enhancing the wall coverage of sinusoidal vessels and contraction, thereby inducing the progression of MAFLD

Intact endothelial cells play a crucial role in vascular tension, as most endogenous vasodilators and constrictors act through mechanisms involving endothelial cells [87]. Endothelial dysfunction, characterized by reduced response to the vasodilator acetylcholine and impaired endothelial NO production, is known to exist in liver cirrhosis and is believed to be one of the causes of cirrhosis-associated portal hypertension [88, 93]. Thromboxane A2 (TXA2) is a vasoconstrictor derived from arachidonic acid through COX and thromboxane synthase, and acts through receptors on smooth muscle cells and HSCs. It is also related to the pathogenesis of increased intrahepatic resistance in liver cirrhosis [95, 96]. The overexpression of COX and the increase of TXA2 and ET-1 production in the perfusion model of MAFLD rats resulted in sinusoidal curve, hemodynamic changes and increased vasoconstriction [97]. Equally important, steatosis animals showed significant changes in liver microvascular structure, and the classical arrangement of sine waves arranged in parallel and separated by the regular trabeculae of hepatocytes was replaced by completely chaotic irregular and flat vascular patterns. These vessels have many interconnected pipes and the presence of multiple “bubbles”, that is, circular extended sine waves at the blind end. This may represent vascular blockage or local leakage due to normal wall destruction or neovascularization [88]. In addition, by using spontaneous immortalized cell lines of HSC origin, studies have shown that HSCs in liver cirrhosis enhance the wall coverage of sinusoidal vessels, and because of the contractile nature of HSCs, this “pathological sinusoidal remodeling” process further promotes high resistance, contractile sinusoidal angiogenesis [98–100]. In fact, by recruiting and activating HSCs to the vascular wall, and extending the tentacle-like structure that surrounds the vascular lumen and the adjacent LSECs, HSCs have the ability to adjust themself around the vascular lumen in an effective way to achieve these pathological changes [101, 102] (Fig. 3).

## LSECs and MASH

Up to 20 -30% of patients with liver steatosis will develop MASH, and more than 30% of patients with MASH may develop cirrhosis or HCC [103]. In the local immune environment of MASH, in addition to the traditional immune cells such as KCs, dendritic cells, lymphocytes, neutrophils and mast cells, LSECs also play a strong immune function when the liver is under severe pressure [104–106]. LSECs express effective scavenger proteins that can clear waste from the bloodstream, making them highly sensitive sentinel cells in the liver [107–109]. Meanwhile, LSECs have a unique fenestration phenotype, with a window structure that facilitates the recruitment of immune cells from the blood to liver parenchymal cells, all of which are involved in the pathological process of MASH [110].

In physiological conditions and early stages of MAFLD, LSECs are known for its anti-inflammatory effects [111]. However, during NALFD progression, LSECs then acquire pro-inflammatory phenotypes and functions, which in turn exacerbates MASH progression [84]. Pro-inflammatory phenotype of LSECs referred to in this review as LSECs endotheliopathy manifested by the release of pro-inflammatory mediators including cytokines and chemokines, aberrant expressions of adhesion molecules, acquisition of angiogenic properties, the loss of fenestrae and the formation of basement membranes [112]. There are several potential candidate mediators, including products derived from visceral adipose tissue, such as ox-LDL, palmitate and adipokines, that can induce LSECs endotheliopathy. In vitro studies showed that stimulation of LSECs with ox-LDL and palmitate activated NF-kB and TLR-4, respectively [49, 113, 114]. In addition, in the case of metabolic syndrome, the circulating concentrations of several adipokines, including TNF-a and IL-6, increase in the portal vein and may lead to LSECs endotheliopathy [115]. The increase of intestinal permeability and plasma concentration of LPS may also contribute to LSECs endotheliopathy [116–118]. The LSECs endotheliopathy during MASH is characterized by progressive overexpression of adhesion molecules, including intercellular cell adhesion molecule-1, vascular cell adhesion molecule-1 (VCAM-1) and vascular adhesion protein 1, as observed in the MASH mouse model [119–124]. LSECs also produced many pro-inflammatory mediators in MASH, including TNF-α, IL-6, IL-1 and CCL2 [119, 125], after recognizing pro-inflammatory mediators through PRRs and SRs. White blood cells homing to the liver and adhering to LSECs is a key element in the pathogenesis of MASH, and it is also a strictly regulated multi-step process [84]. VCAM-1 is a member of the cell adhesion molecule immunoglobulin superfamily, which is mainly expressed on the surface of endothelial cells and regulates the firm adhesion between leukocytes and endothelial cells [126]. Lipotoxic stress enhances the expression of VCAM-1 in LSECs through MLK3/P38 signaling, while inhibition of VCAM-1 can inhibit adhesion and transendothelial migration of monocytes across LSECs (from wild-type mice fed a high-fat diet and from ob/ob obese mice) and improves liver inflammation [31, 124].

**Fig. 4:**
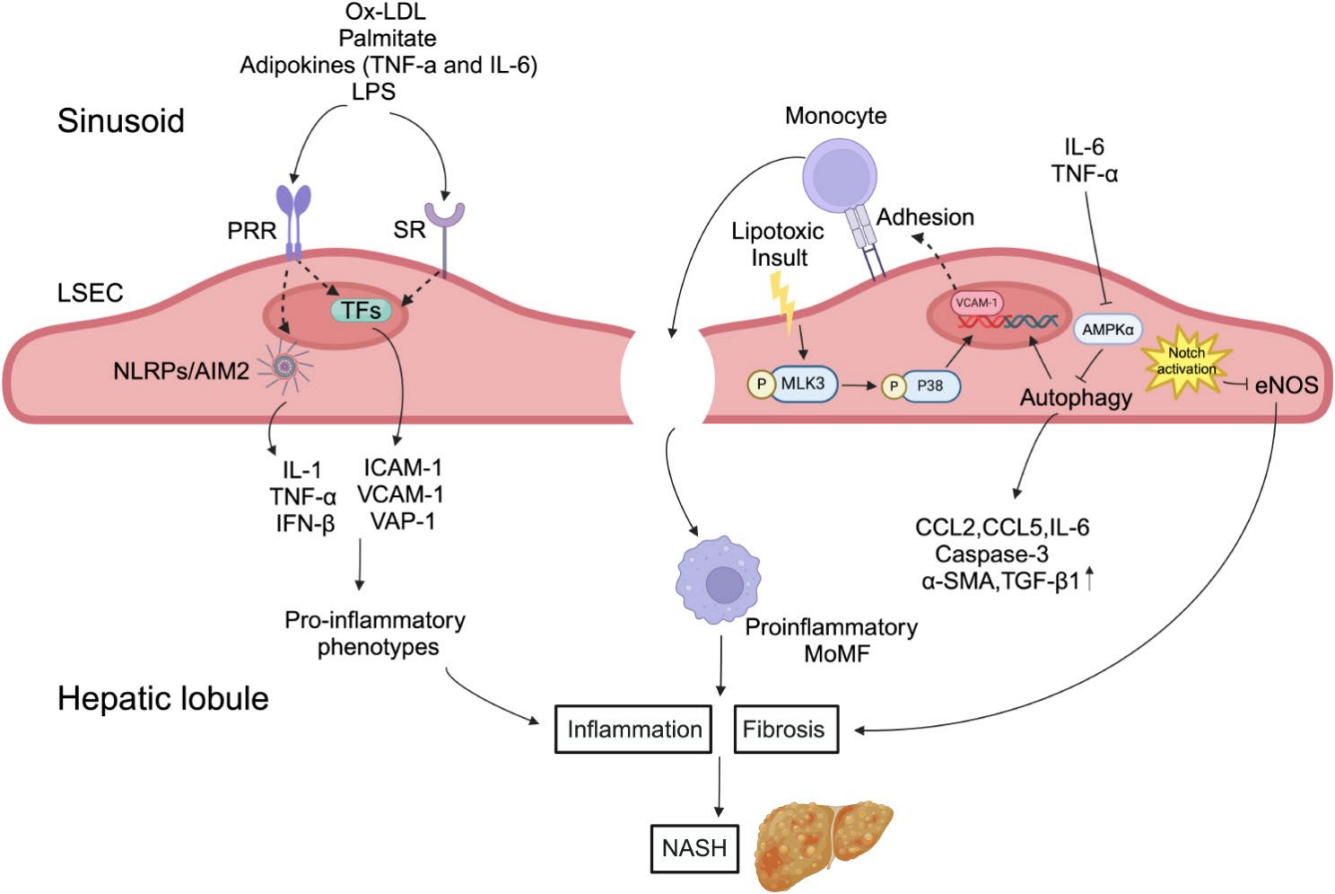
Ox-LDL, palmitate, adipokines (TNF-a and IL-6) and LPS lead to inflammatory phenotypes in LSECs, which induce LSECs to produce pro-inflammatory mediators in MASH, including TNF-α, IL-6, IL-1 and CCL2. Lipotoxic stress can enhance the VCAM-1 expression of LSECs through MLK3/P38 signal transduction, and stimulate monocytes to gather in the liver through fenestrae and activate into a pro-inflammatory state. In addition, IL-6 and TNF-α in local microenvironment down-regulate autophagy and increase the expression of CCL2, CCL5, IL-6, Caspase-3, α-SMA and TGF-β1 by inhibiting AMPKα, and activation of Notch signal in damaged LSECs inhibits eNOS transcription, which aggravates liver inflammation and fibrosis, and then promotes the progression of MASH. MOMF: monocyte-derived macrophages

In addition, MASH is associated with LSECs autophagy deficiency. The number of LSECs with autophagy vacuoles in MASH patients is half of that in normal subjects [127]. In mice fed a high-fat diet or treated with carbon tetrachloride, the LSECs of endothelial autophagy deficiency induced by IL-6 and TNF-α in the liver show upregulation of genes related to inflammatory pathways (CCL2, CCL5, CD68, VCAM-1), hepatocyte apoptosis (lytic Caspase-3) and peri-sinusoidal fibrosis. Consistently, autophagy defects enhance the expression of inflammation-related genes (CCL2, CCL5, IL-6 and VCAM-1), apoptosis (lytic Caspase-3) and endothelial-interstitial transformation characteristics (α-SMA, TGF-β1, COLLA2 expression) by inhibiting AMPKα in LSECs cell lines [127]. At the same time, Notch/eNOS signal pathway plays an important role in the progression of MASH. Notch activation aggravates MASH by inhibiting eNOS transcription in methionine-choline-deficient diet-induced MASH mouse models, while pharmacological activation of eNOS can reduce liver inflammation and lipid deposition caused by Notch activation [128] (Fig. 4).

## LSECs and liver fibrosis

LSECs and HSCs maintain their respective differentiation phenotypes, but the maintenance of LSECs differentiation requires the secretion of VEGF-A by HSCs and hepatocytes to function through the NO/sGC/cGMP pathway and the NO-independent pathway [55]. Meanwhile, differentiated LSECs can prevent HSCs activation and induce aHSC to reverse to resting state, preventing disease progression or promoting fibrosis regression during sustained injury [15]. But when LSECs undergo dedifferentiation or “capillarization” the protective effect disappears [65]. Using rats transplanted with transgenic enhanced green fluorescent protein-positive BM to identify the LSECs mediator that maintains HSCs quiescence. The study shows that capillarization is due to repair of injured LSECs by BM endothelial progenitors that engraft but fail to fully mature. Lack of maturation of BM-derived LSECs is due to cell autonomous pathways that inhibit the nitric oxide pathway [80]. Heparin-binding epidermal growth factor (HB-EGF) is a signal to keep HSCs still [129]. Undifferentiated LSECs cannot shed HB-EGF from the cytoplasmic membrane, thus creating an environment conducive to liver fibrosis [80].

Hh pathway is a key signaling pathway that regulates cell fate decision-making, covering proliferation, differentiation, migration, and apoptosis [130–132]. It plays a crucial role in histogenesis during fetal development [132]. In the process of inducing LSECs spontaneous capillarity in vitro, Hh signal is activated, while inhibition of Hh pathway reduces Hh ligands, mesenchymal genes and capillarization markers, indicating that Hh pathway can regulate LSECs capillarization in vitro. In vivo, Hh signal activation promotes the formation of LSECs tubular structure and the accumulation of hepatic myofibroblasts, while blocking Hh pathway can prevent LSECs capillary formation and liver fibrosis [133, 134]. In addition, studies have shown that after bile duct ligation, the expression of adipocyte-fatty acid binding protein (A-FABP) in mouse LSECs is induced, and the gene ablation or pharmacological inhibition of A-FABP can reduce ligation or carbon tetrachloride-induced liver fibrosis in mice. In terms of mechanism, elevated A-FABP promotes LSECs capillarization by activating Hh signal transduction, which in turn impairs the gatekeeper function of LSECs to HSCs activation. In addition, LSECs-derived A-FABP acts directly on HSCs in a paracrine manner, which enhances the trans-activation of TGF-β1 by activating c-Jun N-terminal kinase (JNK)/c-Jun signal transduction, thus aggravating liver fibrosis induced by HSCs activation [54]. It is suggested that Hh signal pathway is a crucial pathway in liver fibrosis.

Organ specific cytokines derived from LSECs, also known as “vascular secretion factors,” are involved in liver development, homeostasis, and disease pathogenesis [135–137]. LSECs secrete BMP-2/6 to regulate iron metabolism by controlling the secretion of ferritin in hepatocytes [138]. Secreted Wnt-2/9b regulates liver growth and maturation, metabolic liver zoning and liver regeneration [139, 140]. Transcription factor GATA4 is the main regulator of LSECs during early liver development, which controls embryonic stem cell migration and fetal hematopoiesis [47]. The genetic defect of Gata4 in adult mouse LSECs leads to continuous endothelial dedifferentiation and peri-sinusoidal liver fibrosis, which involves downregulation of BMP-2 and Wnt-2 signaling, activation of transcription factor MYC, and de novo expression of hepatic stellate cell activating cytokine platelet-derived growth factor (PDGF) subunit B. This suggests that endothelial GATA4 prevents perisinusoidal liver fibrosis by inhibiting MYC activation and fibrogenic vascular secretion signal transduction at the chromatin level [33, 47]. In addition, the vascular secretion signals of LSECs are correlated with an increase in mechanical tensile strength, making them highly suitable for sensing stiffness and generating vascular secretion programs that regulate liver fibrosis and portal hypertension [141, 142]. Glycolytic enzymes, especially phosphofructokinase 1 isoform P (PFKP), are enriched in specific focal adhesion proteins isolated from gel-fixed LSECs, and stiffness causes PFKP recruitment to plaques, which is parallel to the increase in glycolysis. Mechanically, glycolysis promotes CXCL1 expression through nuclear pore changes and increased NF-kB translocation. The secretion of CXCL1 induces neutrophil infiltration, which in turn promotes early liver fibrosis and portal hypertension [141, 143–145].

**Fig. 5:**
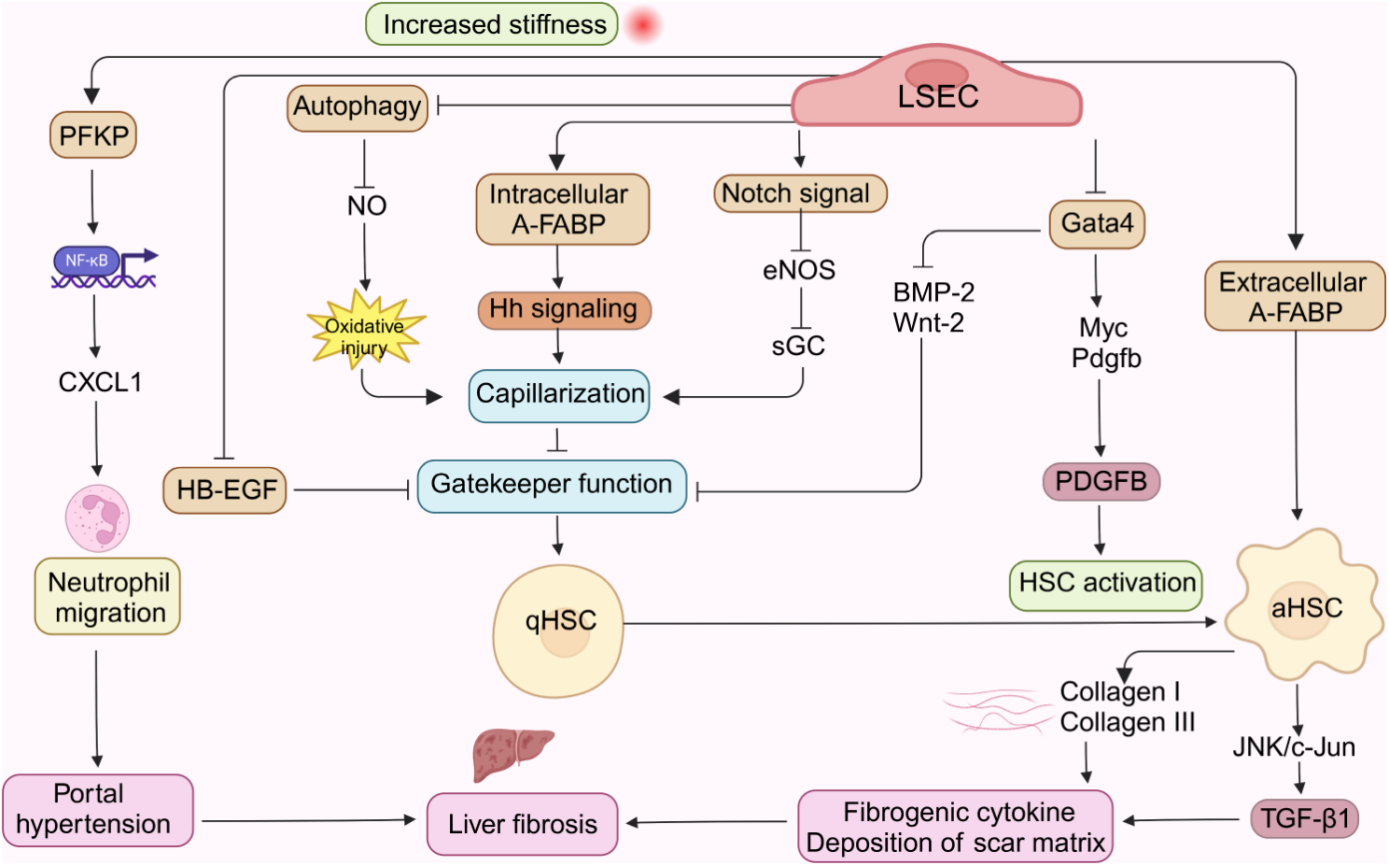
LSECs related pathological mechanism of liver fibrosis. When the stiffness of LSECs increases due to mechanical stretching, PFKP recruits specific macular adhesion proteins, and the increased glycolysis promotes CXCL1 expression and neutrophil infiltration, which in turn promote hepatic portal hypertension and early fibrosis. HB-EGF is the signal to keep HSCs still, but undifferentiated LSECs cannot shed HB-EGF from the cytoplasmic membrane, thus creating an environment conducive to HSCs activation. In addition, the down-regulation of LSECs autophagy caused by chronic liver injury leads to cellular dysfunction and the decrease of intrahepatic NO, leading to oxidative stress in vivo. Elevated A-FABP promotes the capillarization of LSECs by activating Hh signal transduction, hence impairing the gatekeeper function of LSECs. The derived A-FABP also acts directly on HSCs in a paracrine manner, which enhances the transactivation of TGF-β1 by activating JNK/c-Jun signal transduction, thus aggravating liver fibrosis caused by HSCs activation. Notch signal of endothelial cells plays an important role in the regulation of liver homeostasis. Endothelial Notch activation destroys liver homeostasis by weakening eNOS/sGC signal transduction, resulting in a decrease in fenestration and an increase in BM. In addition, Gata4 inhibition leads to down-regulation of BMP-2 and Wnt-2 signals, activation of MYC and ab initio expression of PDGFB, which in turn aggravate the activation of HSCs and perisinusoidal liver fibrosis

Notch signal of endothelial cells plays an important role in the regulation of liver homeostasis [146–148]. In current research, endothelial Notch activation destroys liver homeostasis by weakening eNOS/sGC signal transduction, resulting in reduced fenestration, increased BM and increased liver fibrosis, while pharmacological activation of sGC can reverse the phenotype of LSECs dedifferentiation [149]. In addition, autophagy maintains LSECs homeostasis, and the pharmacological or genetic downregulation of endothelial autophagy can induce cell dysfunction and decrease of intrahepatic NO, leading to oxidative stress in the body and aggravating liver fibrosis [127, 150]. In the early stage of liver diseases, autophagy flux helps to maintain endothelial phenotype and protect LSECs from oxidative stress. Therefore, selectively enhancing autophagy of LSECs in the early stage may be an attractive method to change the course of the disease and prevent fibrosis progression [150] (Fig. 5).

## LSECs and HCC

MAFLD is already the fastest growing cause of HCC in the USA, France and the UK [9]. Inflammation is a key factor in the progression of HCC [151, 152]. Liver tumor is the result of proliferative and invasive characteristics of precancerous lesions, which are caused by genetic and epigenetic changes developed in the context of persistent inflammatory liver damage [153]. HCC induces phenotypic changes in peripheral LSECs, which helps to reduce the anti-tumor immune response [154]. On the other hand, capillarized LSECs can participate in angiogenesis, coagulation and fibrinolysis events during tumorigenesis [155], indicating the role of LSECs in tumor vascular remodeling during HCC progression [156].

PD-L1 and PD-L2, as well as costimulatory molecules CD80 and CD86, are expressed by LSECs as part of their antigen-presenting function [157]. These structures constitute ligands for immune checkpoint PD1 and cytotoxic T lymphocyte antigen 4 (CTLA4) in T cells, respectively [158–160]. In the interaction, the activation of T cells is inhibited and a state of tolerance differentiation promoted by locally produced IL-10 is obtained [157]. Although LSECs increase the expression of CD151, which regulates the activity of VCAM-1 and cooperates with T cell to recruit [161]. However, the overexpression of PD-L1 in LSECs during HCC [162, 163] leads to the inhibition of T cell function and limits T cell anti-tumor activity [164]. In addition, circulating fatty acid binding protein 4 (FABP4) levels are elevated in non-HCC or MAFLD patients and are associated with liver inflammation and fibrosis [165]. Recently, it has been proven that there may also be a correlation between FABP4 and the progression of HCC. LSECs exposed to high concentrations of glucose, insulin or VEGF-A can induce hepatocyte proliferation by releasing FABP4. In mice fed a high-fat diet, downregulation of FABP4 inhibited the growth of HCC [166]. Therefore, it can be speculated that the FABP4 from LSECs is helpful to the development of HCC [84].

**Fig. 6:**
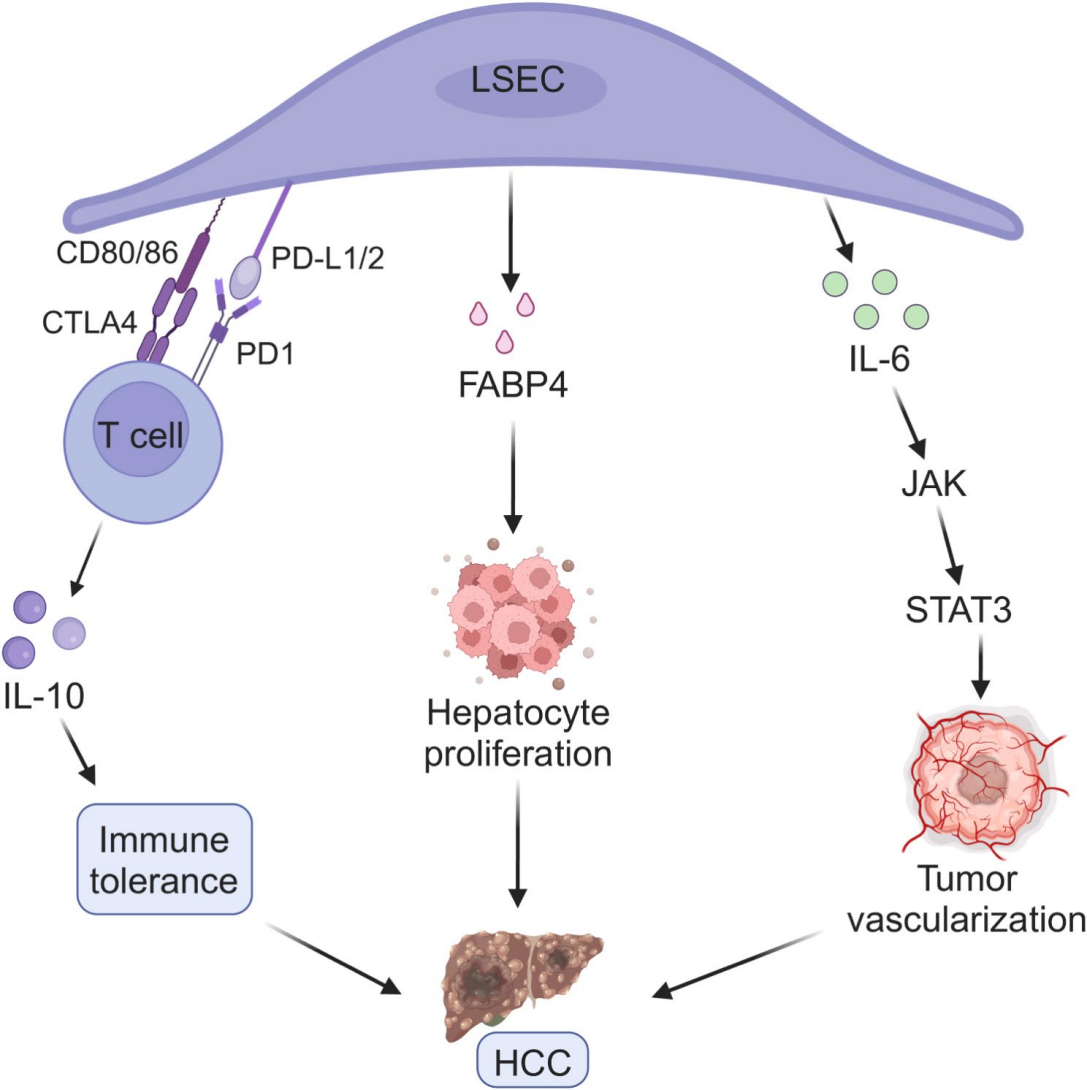
LSECs related pathological mechanism of HCC. The PD-L1/2 and CD80/86 expressed on LSECs constitute the ligands of PD1 and CTLA4 in T cells, respectively. In the interaction, the activation of T cells is inhibited and a state of tolerance differentiation promoted by locally produced IL-10 is obtained. In the microenvironment, LSECs exposed to high concentration of glucose, insulin or VEGF-A can induce hepatocyte proliferation and promote HCC growth by releasing FABP4. In addition, IL-6 and IL-6 receptor secreted by peritumoral endothelial cells and macrophages lead to highly vascularized tumors, which simultaneously aggravate the development of tumor occurrence

In the same line, the peritumoral endothelial cells isolated from HCC patients proliferate more in the culture with IL-6 and soluble IL-6 receptor. IL-6 binds to the IL-6 receptor and then triggers the Janus kinase associated with the receptor, stimulating phosphorylation and activating signal transducer and activator of transcription 3 to initiate downstream angiogenesis, which leads to highly vascularized tumors and development of tumor occurrence. In the process of tumor occurrence, a large number of macrophages exist in the peritumoral liver tissue. IL-6 and IL-6 receptor are secreted by peri-tumor endothelial cells and macrophages, respectively [167, 168]. These data indicate that peritumoral endothelial cells play a major role in the progression of HCC [169] (Fig. 6).

## Treatment of LSECs in liver diseases

The high prevalence and clinical importance of MAFLD have surfaced [170, 171]. However, no effective treatment strategy for MAFLD has been found. Improving diet quality and increasing physical activity are the only management methods available at present [172–176]. From a liver-centered point of view, in order to avoid over-diagnosis and over-treatment, general practitioners should focus on the diagnosis and treatment of MASH patients with moderate to severe fibrosis [177]. Cell adhesion molecules such as integrin β 1 (ITG β 1) and VCAM-1 play a key role in the development of MASH. Under lipotoxic stress, ITG β 1 is released from hepatocytes as a cargo of extracellular vesicles and mediates the adhesion of monocytes to LSECs, which is an important step in liver inflammation [178]. VCAM-1 is released from LSECs, increasing the number and adhesion of proinflammatory monocytes in the liver, thereby promoting the progress of MASH [179]. In MASH mouse model, blocking ITG β 1 or VCAM-1 can reduce liver inflammation, injury and fibrosis [31, 180]. Therefore, the inhibition of cell adhesion molecules may be a new therapeutic strategy for MASH [31, 178].

Statins up-regulate Kruppel-like factor 2 (KLF2) in LSECs [181]. KLF2 induces vascular protection and HSCs inactivation through paracrine mechanism mediated by KLF2/NO/sGC signal, thus improving hepatic endothelial function and hepatic fibrosis in the experimental model of liver cirrhosis [182]. Peroxisome proliferator-activated receptor α (PPARα) is a ligand-activated transcription factor that regulates genes related to vascular tension, oxidative stress and fibrogenesis, and is related to the development of and portal hypertension [183, 184]. Fenofibrate can reduce the production of COX-1 and thromboxane in rats, increase the bioavailability of NO in LSECs, reduce portal pressure and hepatic fibrosis in cirrhotic rats, and improve hepatic endothelial dysfunction by activating PPAR α [185, 186]. A fairly short-term (6-month) study conducted by Belfort and colleagues confirmed that pioglitazone can reverse MASH in a short period of time [187]. In addition, a meta-analysis of all available randomized trials showed that pioglitazone was beneficial in patients with advanced fibrosis [187–190].These findings confirm the hypothesis that the improvement of metabolic damage may help slow the progression of fibrosis [191]. PX20606 (PX), a novel non-steroidal selective farnesate X receptor (FXR) agonist, can induce hepatic sinusoidal vasodilation (CTH, DDAH1, eNOS and GCH1 up-regulated) and reduce intrahepatic vasoconstriction (ET-1 and p-Moesin down-regulated). In liver cirrhosis, PX can improve endothelial dysfunction and normalize the overexpression of VEGF-A, PDGF and angiopoietin. In human LSECs, PX treatment can significantly reduce portal vein pressure by inhibiting hepatic sinusoid remodeling [192].

**Fig. 7:**
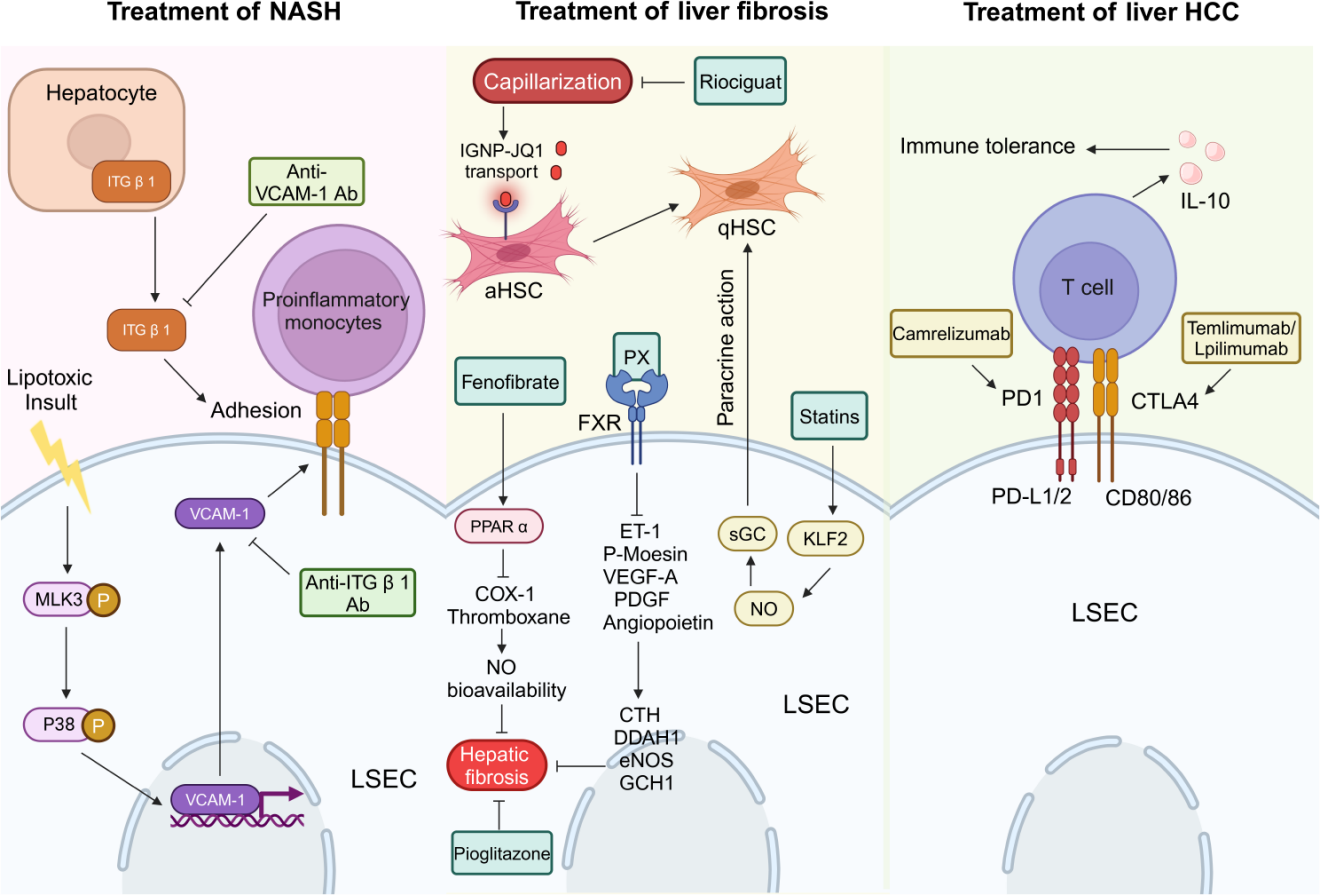
Treatment of LSECs in liver diseases. ITG β1 from hepatocytes and VCAM-1 from LSECs mediate the recruitment and adhesion of monocytes to LSECs in the development of MASH, so blocking ITG β1 or VCAM-1 can reduce liver inflammation and may be a new therapeutic strategy for MASH. Statins up-regulate KLF2 in LSECs. KLF2 induces HSCs inactivation through paracrine mechanism mediated by KLF2/NO/sGC signal, thus improving hepatic endothelial function and hepatic fibrosis. Fenofibrate can reduce the production of COX-1 and thromboxane, increase the bioavailability of NO in LSECs, reduce hepatic fibrosis and improve hepatic endothelial dysfunction by activating PPARα. In addition, pioglitazone is also beneficial in patients with advanced fibrosis. PX is a novel non-steroidal selective FXR agonist, which can induce hepatic sinusoidal vasodilation (CTH, DDAH1, eNOS and GCH1 up-regulated) and reduce intrahepatic vasoconstriction (ET-1, p-Moesin, VEGF-A, PDGF and angiopoietin down-regulated), thus improving endothelial dysfunction. Newly discovered, Riociguat can reverse sinusoidal capillarization and maintain relatively normal LSECs porosity, thereby promoting the transport of IGNP-JQ1 through the sinusoidal endothelial wall, which is then selectively absorbed by aHSCs in the Disse space to reduce collagen deposition in the liver. Regarding the treatment of HCC, Camrelizumab and Temlimumab /Lpilimumab can target PD1 and CTLA4 on T cells separately, thus inhibiting the secretion of IL-10 and immune tolerance

During the development of liver fibrosis, capillarization of LSECs restricts the exchange of substances between blood and the Disse space, further accelerating the activation of HSCs and the process of fibrosis [193, 194].The Disse space therapy drugs are often ignored, which remains the main bottleneck of HSCs targeted therapy for liver fibrosis. However, this challenge can be preliminarily addressed by pretreatment with soluble guanylate cyclase stimulator, Riociguat, and then targeted delivery of insulin growth factor 2 receptor-mediated antifibrotic drug JQ1 by peptide nanoparticles (IGNP). Riociguat can reverse sinusoidal capillarization and maintain relatively normal LSECs porosity, thereby promoting the transport of IGNP-JQ1 through the sinusoidal endothelial wall and its accumulation in the Disse space. IGNP-JQ1 is then selectively absorbed by aHSCs, inhibiting its proliferation and reducing collagen deposition in the liver. This combination strategy can significantly eliminate the fibrosis induced by carbon tetrachloride or methionine-deficient diet in MASH mice. The strategy of restoring the LSECs fenestration through Riociguat represents a promising treatment for liver fibrosis [195, 196] (Fig. 7).

Blocking drugs targeting new angiogenesis, cell proliferation, cell survival or cell movement signaling pathways is a consideration strategy in liver tumor treatment [197]. In clinical trials of HCC (Table 1), first-line drugs Sorafenib [198] or Lenvatinib [199], as well as second-line drugs Regorafenib [200] or Cabozantinib [201], have been shown to improve clinical outcomes [202]. In addition, LSECs express ligands related to immune checkpoints that inhibit or stimulate T cell responses. Therefore, monoclonal antibodies targeting PD1, Camrelizumab, can also alleviate objective symptoms in patients and improve survival rate [203, 204]. Monoclonal antibodies targeting CTLA4, such as Temlimumab and Lpilimumab, are being studied in HCC patients, and both drugs are currently in phase III clinical trials (NCT03412773) [205].

**Table 1:** Summary of therapies for MAFLD related liver diseases targeting LSECs

Study population	Treatment	Results	Ref.
Diet-induced MASH mice	Anti-VCAM-1 Ab/ VCAM-1 inhibitor AGI-1067	Reduce the number of proinflammatory monocytes in the liver and relieve MASH in mice	[33]
Diet-induced MASH mice	Anti-ITG β 1 Ab	Reduce the number of proinflammatory monocytes in the liver and relieve MASH in mice	[177]
CCl4 cirrhotic rats	Atorvastatin/Mevastatin/ Simvastatin/Lovastatin	Up-regulation of KLF2 to promote vascular protection and HSCs inactivation	[181]
CCl4 cirrhotic rats	Fenofibrate (25 mg/kg/d, oral, 7d)	Improve portal hypertension and liver fibrosis	[184]
Cirrhotic rats (thioacetamide/common bile duct ligation)	Pan-PPAR agonist Lanifibranor (100 mg/kg/d, oral, 14d)	Improve portal hypertension and liver fibrosis	[185]
Patients with impaired glucose tolerance/T2DM complicated with MASH	Pioglitazone (45 mg/d, oral, 6 m)	Improve blood glucose, liver steatosis, ballooning necrosis and inflammation	[186]
Patients with prediabetes/T2DM complicated with MASH	Pioglitazone (45 mg/d, oral, 18 m)	Relieve insulin resistance, liver steatosis, MASH and fibrosis	[187]
MASH patients	Pioglitazone (30 mg/d, oral, 12 m)	Improve blood glucose, hepatocyte injury and fibrosis	[188]
MASH patients	Pioglitazone (30 mg/d, oral, 96w)	Reduce hepatocyte injury, steatosis and lobular inflammation	[189]
Portal hypertensive rats	FXR agonist PX20606 (10 mg/kg, intragastric administration, 14w)	Reduce hepatic fibrosis, vascular remodeling and hepatic sinusoid dysfunction, and improve portal hypertension	[191]
CCl4-induced fibrosis mice/MASH mice lacking methionine-choline diet	IGNP-JQ1	Reverse capillarization and eliminate liver Fibrosis	[195]
HCC patients	Sorafenib (400 mg/d, oral)	Extend the median survival time by 3 months	[197]
HCC patients	Lenvatinib (12 mg/d for body weight ≥ 60 kg, 8 mg/d for body weight < 60 kg, oral)	Extend the overall survival time with good safety and tolerance	[198]
HCC patients	Regorafenib (160 mg/d, oral)	Improve overall survival and median survival	[199]
HCC patients	Cabozantinib (100 mg/d, oral, 12w)	Objective symptom relief, tumor regression and decrease of alpha-fetoprotein	[200]
HCC patients	Camrelizumab (3 mg/kg, intravenous, 6 m)	Improve overall survival rate	[203]

## Conclusions

As the gatekeeper of liver dynamic balance, the importance of LSECs in MAFLD and derived diseases is often ignored. This is closely related to the characteristics of these cells, as the research conclusions of LSECs are partly based on in vitro experiments. However, LSECs rapidly dedifferentiates after isolation and cultivation, resulting in differences between the in vitro model and the actual pathological mechanism. New experimental methods, such as adding specific matrix and biomechanical stimulation, are needed to maintain the differentiation characteristics of LSECs in vitro and improve the credibility of the model, so as to explore the potential of LSECs in the prevention and treatment of MAFLD.

## Data Availability

No datasets were generated or analysed during the current study.

## Abbreviations

LSECs: Liver sinusoidal endothelial cells
MAFLD: Metabolic dysfunction-associated fatty liver disease
MASH: Metabolic dysfunction-associated steatohepatitis
NAFLD: Non-alcoholic fatty liver disease
NASH: Non-alcoholic steatohepatitis
HCC: Hepatocellular carcinoma
HSCs: Hepatic stellate cells
KCs: Kupffer cells
BM: Basement membrane
LDL: Low-density lipoprotein
ox-LDL: Oxidized low-density lipoprotein
ROS: Reactive oxygen species
ET-1: Endothelin-1
eNOS: Endothelial nitric oxide synthase
LPS: Lipopolysaccharide
Hh: Hedgehog
iNOS: Inducible nitric oxide synthase
VEGF: Vascular endothelial growth factor
sGC: Soluble guanylate cyclase
cGMP: Cyclic guanosinc monophosphate
PKG: Protein kinase G
BMP9: Bone morphogenetic protein 9
COX: Cyclooxygenase
MHC: Major histocompatibility complex
PD-L1: Programmed death ligand 1
PD-1: Programmed death 1
aHSC: Activated HSC
qHSC: Quiet HSC
VLDL: Very-low-density lipoprotein
TC: Total cholesterol
TG: Triglycerides
TXA2: Thromboxane A2
VCAM-1: Vascular cell adhesion molecule-1
MOMF: Monocyte-derived macrophages
HB-EGF: Heparin-binding epidermal growth factor
A-FABP: Adipocyte-fatty acid binding protein
JNK: Jun N-terminal kinase
PDGF: Platelet-derived growth factor
PFKP: Phosphofructokinase 1 isoform P
CTLA4: Cytotoxic T lymphocyte antigen 4
FABP4: Fatty acid binding protein 4
ITG β 1: Integrin β 1
KLF2: Kruppel-like factor 2
PPARα: Peroxisome proliferator-activated receptorα
PX: PX20606
FXR: Farnesate X receptor
IGNP: Peptide nanoparticles
